# SkewC: Identifying cells with skewed gene body coverage in single-cell RNA sequencing data

**DOI:** 10.1016/j.isci.2022.103777

**Published:** 2022-01-15

**Authors:** Imad Abugessaisa, Akira Hasegawa, Shuhei Noguchi, Melissa Cardon, Kazuhide Watanabe, Masataka Takahashi, Harukazu Suzuki, Shintaro Katayama, Juha Kere, Takeya Kasukawa

**Affiliations:** 1 Laboratory for Large-Scale Biomedical Data Technology, RIKEN Center for Integrative Medical Sciences, 1-7-22 Suehiro-cho, Tsurumi-ku, Yokohama City, Kanagawa, 230-0045, Japan; 2 Laboratory for Cellular Function Conversion Technology, RIKEN Center for Integrative Medical Sciences, 1-7-22 Suehiro-cho, Tsurumi-ku, Yokohama City, Kanagawa, 230-0045, Japan; 3 Folkhälsan Research Center, Topeliuksenkatu 20, 00250 Helsinki, Finland; 4 Department of Biosciences and Nutrition, Karolinska Institutet, 141 83 Huddinge, Sweden; 5 Stem Cells and Metabolism Research Program, University of Helsinki, P.O. Box 4 (Yliopistonkatu 3), Helsinki, Finland; 6 Institute for Protein Research, Osaka University, Suita, Osaka 565-0871, Japan

**Keywords:** Biological sciences, Cell biology, Biocomputational method, Biological sciences research methodologies

## Abstract

The analysis and interpretation of single-cell RNA sequencing (scRNA-seq) experiments are compromised by the presence of poor-quality cells. For meaningful analyses, such poor-quality cells should be excluded as they introduce noise in the data. We introduce SkewC, a quality-assessment tool, to identify skewed cells in scRNA-seq experiments. The tool’s methodology is based on the assessment of gene coverage for each cell, and its skewness as a quality measure; the gene body coverage is a unique characteristic for each protocol, and different protocols yield highly different coverage profiles. This tool is designed to avoid misclustering or false clusters by identifying, isolating, and removing cells with skewed gene body coverage profiles. SkewC is capable of processing any type of scRNA-seq dataset, regardless of the protocol. We envision SkewC as a distinctive QC method to be incorporated into scRNA-seq QC processing to preclude the possibility of scRNA-seq data misinterpretation.

## Highlights

• We developed a quality assessment method applicable to single-cell RNA-Seq data
• SkewC relies on the use of gene coverage profile of cells as a quality measure
• SkewC reveals two classes of cells: typical cells and skewed cells
• Typical with prototypical coverage profiles, and skewed with skewed profiles

## Graphical abstract

**Figure:**
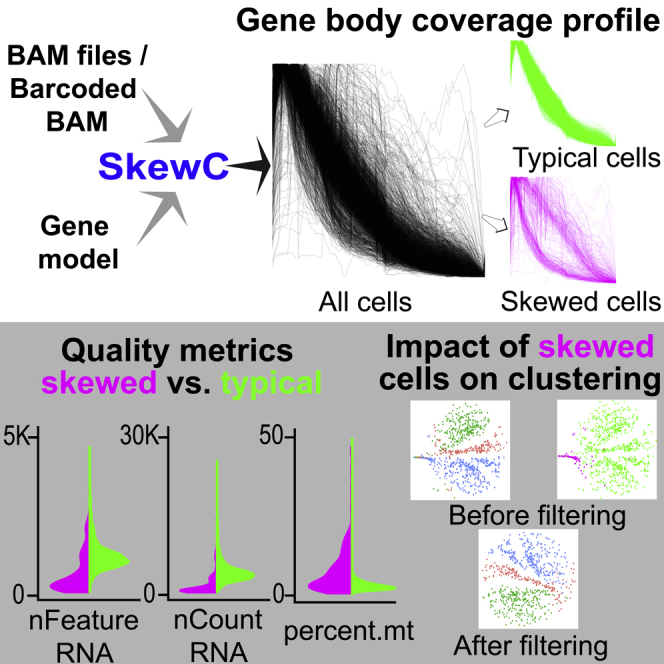

## Introduction

Recent advances in scRNA-seq technologies have enabled new discoveries and insights into the biology of cells (Töhönen et al., 2015; Mathys et al., 2019). These new analyses have been used to profile gene expression of individual cells under different biological conditions, identify new cell types, providing new insights about varied biological processes (Giladi and Amit, 2018).

Quality measures and quality control (QC) methods aim to provide confidence in the quality of the dataset by assuring the robustness, reproducibility, and high quality of any experimental study. In scRNA-seq, the failure to provide adequate quality assessment might lead to the presence of poor-quality cells (dead or live cells (Islam et al., 2014). Without QC steps, incorrect interpretation or compromised resolution is possible, resulting from misclustering errors, propagation of specific cell type population, or insufficient sensitivity to detect differentially expressed genes (DE-Gs). Researchers looking to classify new cell types, and those who do not take the correct QC precautions, can be trapped by misinterpreting a cluster of poor-quality cells as a new cell type.

Moreover, in scRNA-seq, some standard QC metrics utilized in bulk RNA-seq protocols cannot be acquired (e.g., the integrity of RNA, RIN value). As well as this, the quality of each sample can be more variable owing to higher technical and biological variation at the single-cell resolution or variation encountered from differences among scRNA-seq experimental procedures. The variation from experimental procedures includes differences in cell capture methods and sequencing protocols, and the exclusion of cells with failed reactions. Furthermore, cell capture techniques might expose individual cells to stress leading to cell death. Cell capture sites may contain debris, from broken cells, or contain multiple cells instead of a single cell. A single-cell reaction may also simply fail (e.g., in reverse transcription).

As reported in several publications (Islam et al., 2014; Treutlein et al., 2014; Ilicic et al., 2016; Brennecke et al., 2013; Xue et al., 2013; Trapnell et al., 2014), the identification of single cells with poor quality is challenging, primarily as they may represent a large population of cells, and may not be limited only to dead cells. Identifying poor-quality cells experimentally (e.g., microscopic techniques or cell staining) is laborious and involves further manipulations, possibly affecting the transcriptome. Computationally, several tools and methods have been developed to identify poor-quality cells (Treutlein et al., 2014; Ilicic et al., 2016; Brennecke et al., 2013; Trapnell et al., 2014). The first generation of these methods assess quality using various metrics, such as sequence read counts, number of expressed genes, gene expression patterns to detect outliers, library sizes, or total unique molecular identifier (UMI) counts (Islam et al., 2014; Brennecke et al., 2013; Trapnell et al., 2014; Lun et al., 2019). The second generation of methods assesses quality using various known quality metrics as well as a threshold decided by machine-learning methods (Ilicic et al., 2016). These methods are based on existing approaches used in bulk RNA-seq QC and analysis. Therefore, they ignore unique characteristics of scRNA-seq experiments, variation of methodology, and the quality properties of individual cells, in comparison to a bulk RNA-seq sample. This results in limitations, both in terms of the classification result and in implementation (Ilicic et al., 2016). The scRNA-tools database (Zappia et al., 2018) gives a full list of the currently available tools for scRNA-seq QC. Here we consider such limitations and introduce SkewC method that are able to segregate cells that are potential of poor quality, owing to their skewed gene body coverage, from good quality cells in any type of scRNA-seq dataset.

## Results

### Wide discrepancies in gene body coverage among scRNA-seq protocols

We computed and analyzed the gene body coverage of different scRNA-seq protocols (Figure 1) (see Method details). Remarkably, gene body coverage shows wide differences among the datasets generated by different scRNA-seq protocols (Figures 1A and 1B; Panels A and B in Figures S1–S7).

**Figure 1:**
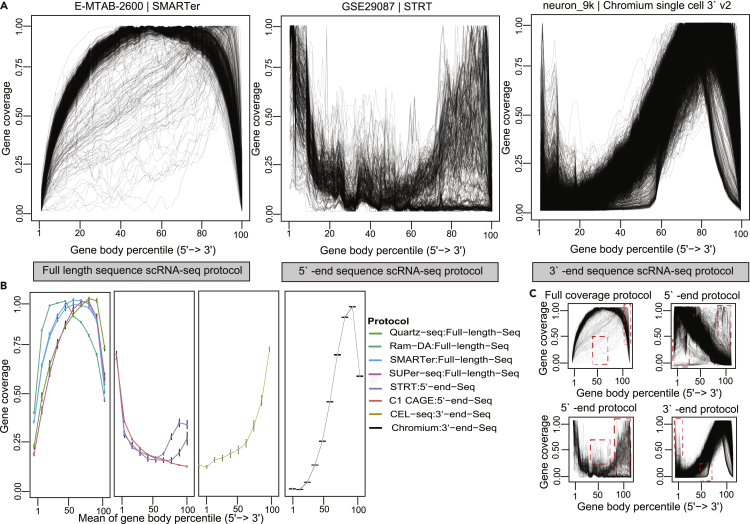
scRNA-seq gene body coverage skewness and skewness distribution Three scRNA-seq datasets. (A) Distribution of mapped reads (tags) across genes. Each panel shows the gene body coverage percentile per dataset. The x axis represents the gene body from 5′ end to 3′ end scaled from 0 to 100, and the y axis denotes gene coverage (0–1). lThe plot of the dataset (ArrayExpress: E-MTAB-2600) generated by SMARTer protocol (full-length sequence) contains cells with low coverage in the middle of the gene region and cells with high coverage in the 3′ -end of the gene region. Although the dataset (NCBI GEO:GSE29087) generated by STRT (5′ -end sequence) contains cells with high coverage both in the middle and the 3′ -end region of the gene. The third dataset from 10x Genomics generated by single-cell 3′ -end protocol contains cells with high coverage in the 5′ -end region of the gene and cells with low coverage in the middle of the gene. (B) Mean of the gene body coverage for different scRNA-seq methods. Error bars represents the standard error of the mean (SEM). (C) Skewness and bias in gene body coverage for cells highlighted with a red dashed box.

We investigated the pattern of the gene body coverage for each cell in individual datasets. From visual inspection of the gene body coverage profile, we can classify them into two sets of gene body coverage patterns. The first one shows a well clustered (i.e., typical) gene body coverage distribution within the single dataset. The second shows a skewed gene body coverage distribution within the single dataset. The skewness in the distribution could be observed in almost all datasets we investigated. There were 3 observable instances of skewness. In one instance, there was higher bias toward the 3′ end of the gene body despite the dataset being created from a 5′ end sequence protocol or full-length sequence protocol, and vice versa (Figure 1C). The tag-based sequencing of 5′ or 3′ end methods should have peaks on gene coverage plots at the 5′ or 3′ end of the gene, respectively, with low/no coverage in the middle region and the opposite ends of the gene body from where the tags are used. However, in the second type, there was higher coverage in the middle of the gene for 5′ and -3′ end sequence protocols, with respect to the full-length sequencing protocols. In the third type, there was low coverage in the middle of the gene for full-length sequence protocols. These variations (biases) and skewness in the gene body coverage among individual cells may subsequently affect the expression profiles of those single cells. If so, the treatment of these skewed cells should be considered when using scRNA-seq data for downstream analyses.

The computed gene body coverage matrix and plots for all datasets are available for download from http://single-cell.riken.jp/Database_Files/gene_body_coverage/

### SkewC segregates typical and potential poor-quality cells

**Figure 2:**
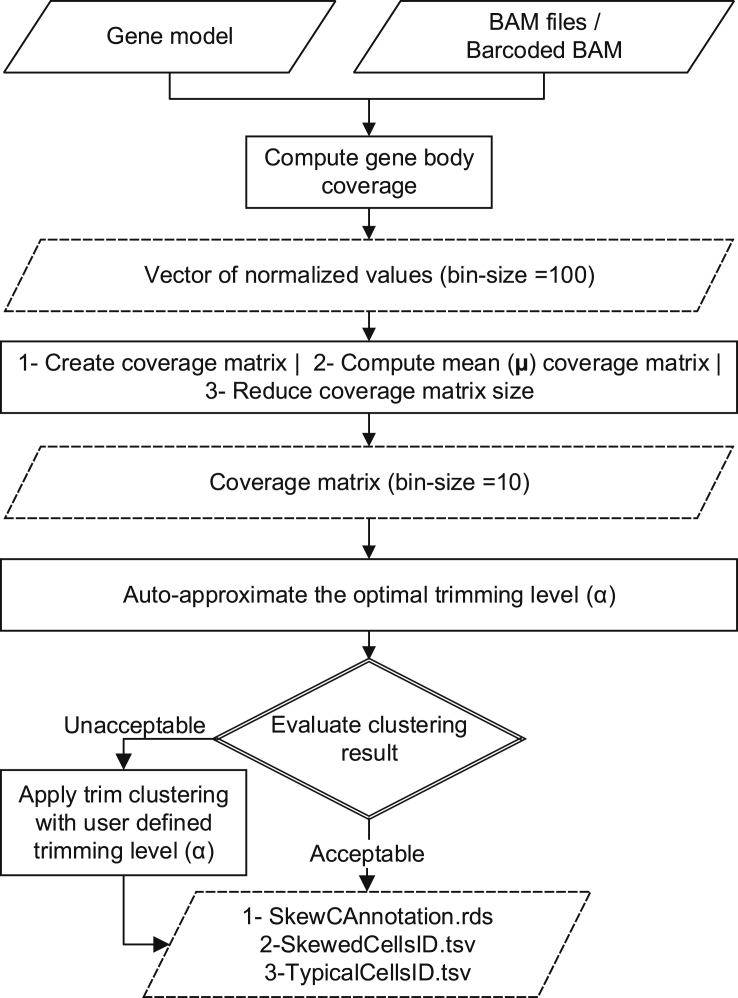
Overview of SkewC workflow The figure illustrates the SkewC workflow and implementation to discriminate skewed cells with skewed gene coverage distribution. The circle numbers callout points to the main inputs, processing, and outputs of SkewC. SkewC inputs are the gene model in. bed format and the aligned reads in BAM format per each cell. For scRNA-seq dataset generated by 10x Genomics libraries, the input to SkewC is the postsorted BAM file together with the cell barcode text file. SkewC bash command to supply the inputs (0_split10XbyBarcode.sh) the batch split the postsorted BAM file into individual BAM files. Compute gene body coverage for each cell. SkewC batch script 1_geneBodyCoverage.sh used to compute gene body coverage and produce a text file.r which contains vector of normalized values. The normalized values are stored as a matrix (coverage matrix with bin size = 100), the coverage matrix should be processed by computing the mean of the coverage matrix and reduce the bin size to be 10. The mean coverage matrix used as in put for the batch script 2_SkewC.sh, which use the trimming clustering function in R (tclust) to cluster the coverage matrix, the script designed to auto approximate the optimal trimming level alpha (**α**) and select the clustering result with optimal alpha (**α**). Other option is to apply the trim clustering with user-defined trimming level alpha (**α**). The output provided in different formats, two text files each with the list of the typical and skewed cells. The other format was R data frame object SkewCAnnotation.rds. The list of annotated single-cells can be added to the R Bioconductor SingleCellExperiment Class or Seurat R package object to be used in QC for filtering skewed cells from analysis.

**Figure 3:**
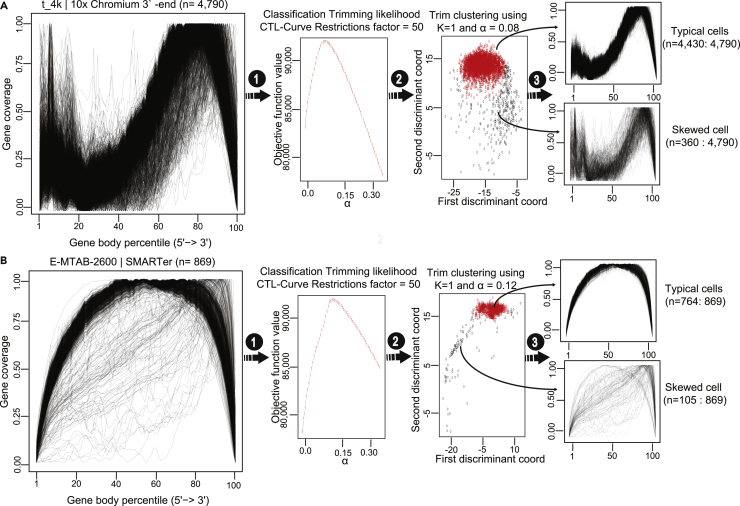
Classification of the typical and skewed coverage distribution cells (A and B) Application of SkewC in two different datasets. The gene body coverage matrix is computed and visualized left chart, (1) the gene body coverage matrix used as input to compute the optimum alpha value. The middle chart shows the CTL-curve with optimal alpha value. (2) The selected alpha value used as input for trim clustering on the coverage matrix. A proportion of the most outlying observations is trimmed (the skewed cells). (3) This resulted in two sets of cells: typical cells (with normal gene coverage) and skewed cells (with skewed coverage distribution).

The results from the gene body coverage analysis discriminate two classes of cells, referred to as typical cells and skewed cells, seen even in one dataset. A method is required to segregate typical cells and skewed cells in any scRNA-seq dataset to avoid biases and large variation. To classify the cells as typical or skewed, we developed an algorithm (SkewC) (Figure 2). We applied the method systematically to all datasets in this study (Figures 3A and 3B; and figures for other datasets available at http://single-cell.riken.jp/SkewC_Online_Suppl_Figs/Classification_of_typical_and_skewed_single_cells/). When applying our method to all datasets, two distinct clusters of cells were visible (middle charts of the figures) corresponding to the two classes mentioned here.

The proper ratio of the typical cells to skewed cells was calculated by the SkewC algorithm, this ratio varied between different datasets. For example, the mouse PBMC dataset had a total number of 8500 single cells, 7904 of which were typical single cells and 596 were skewed single cells. On the other hand, the human PBMC dataset, with a total number of 8258 single cells, showed 7101 typical cells and 1157 skewed cells.

### Expression of housekeeping genes varied between typical and skewed cells

**Figure 4:**
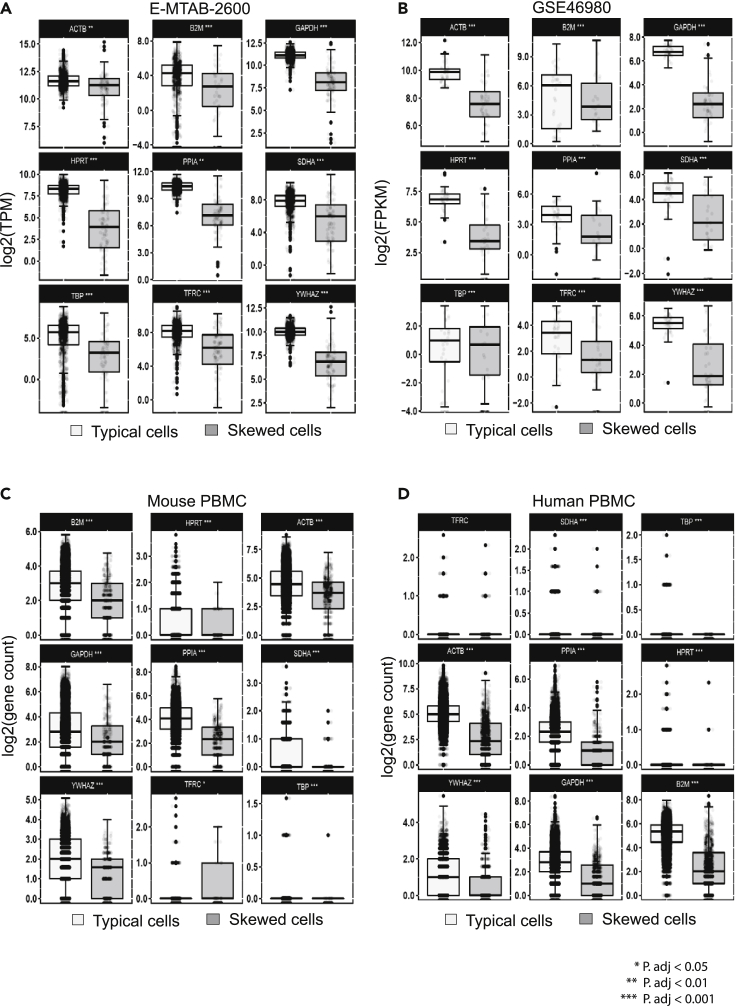
Variation in gene expression of housekeeping genes between typical and skewed cells (A–D) Boxplot of the expression of the house-keeping genes of the typical vs. skewed cells for four datasets. Error bar represents the standard error of the mean (SEM).

To investigate the difference between typical and skewed cells in the resulting gene expression, we compared the normalized expression of a set of housekeeping genes (HKGs) in the typical cells versus the skewed cells (Figures 4A–4D). The boxplots showed distinct differences in the variability in gene expression of the HKGs between the two classes of cells (with very small, adjusted p values).

### Validation of SkewC and the biological features of the skewed cells

**Figure 5:**
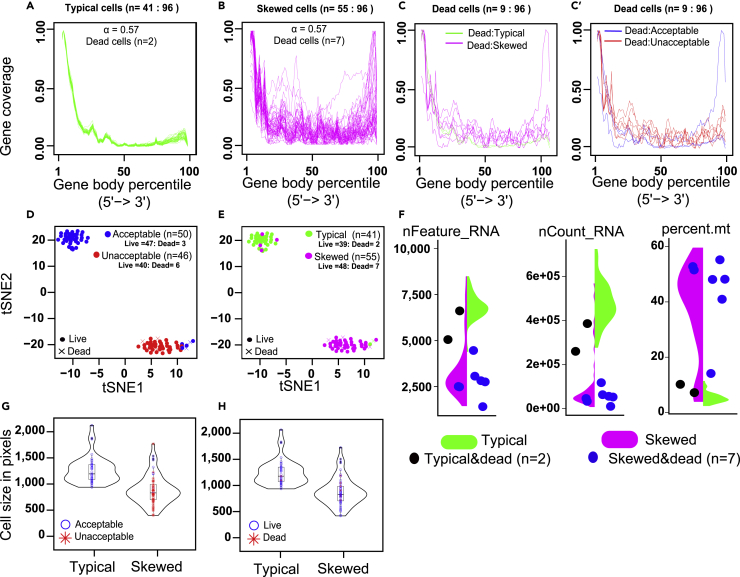
Validation of SkewC method and biological features of skewed cell using (NCBI GEO: GSE46980) We used an existing experimentally validated dataset to validate SkewC. The dataset used was (NCBI GEO: GSE46980). (A–C) the gene body coverage plots for typical, skewed, and all dead cells. The gene body coverage of the dead cells colored based on the SkewC annotation (C) and the (NCBI GEO: GSE46980) authors’ annotation (C′). (D and E) *t*-SNE illustrating the clustering of cells as annotated by the (NCBI GEO: GSE46980) dataset authors (Acceptable and Unacceptable) (D) and typical and skewed cells (E). Dead and live cells are highlighted as well in *t*-SNE. (F) Violin plots showing Seurat QC metrics. The violin plot split by the SkewC annotation and dead cells are shown as black and blue circles. (G) The distribution of cell size between typical and skewed cells, acceptable and unacceptable are highlighted. (H) The distribution of cell size between typical and skewed cells, dead and live is highlighted.

To validate SkewC, we used the mESCs dataset (NCBI GEO: GSE46980 (Islam et al., 2014)). The authors of the dataset classified the 96 cells in their experiment as having either acceptable quality (n = 50), unacceptable quality (n = 46), or as dead cells; identified putatively by the authors using Red Fixable Dead Cell Stain. This resulted in (n = 9) dead cells distributed between the acceptable (n = 3) and unacceptable (n = 6) cell groups. We applied SkewC using all 96 cells. SkewC was run using the Auto Alpha estimation (see Method details), which resulted in typical cells (n = 80) and skewed cells (n = 16) (Figure S8), then we re-ran SkewC with manual optimal alpha value approximation and obtained typical cells (n = 41) and skewed cells (n = 55). The gene body coverage of the typical cells (Figure 5A), skewed cells (Figure 5B), and dead cells (Figure 5C) show different patterns of gene body coverage between the different cell groups. The *t*-distributed stochastic neighbor embedding (*t*-SNE) (Maaten and Hinton, 2008) plot Figure 5D illustrates the clustering of acceptable and unacceptable cells as well as highlighting the dead and live cells. *t*-SNE in Figure 5E shows the clustering of the typical and skewed cells, again we highlighted live and dead cells. Figure 5E shows a clear distinction between typical and skewed cells. SkewC reduced the number of acceptable cells from n = 50 to n = 41 and increased unacceptable cells from n = 46 to n = 55. This indicated that the standard QC procedures currently used in the scRNA-seq analysis were insufficient to discover all cells with potential poor quality.

We performed differential gene expression analysis between the typical and skewed cells. The clustering of the top 100 most variable genes across cells is illustrated in the heatmap (Figure S9); typical and skewed cells are clustered separately based on the gene expression of the top 100 expressed genes. To further evaluate the quality of typical versus skewed cells, we used the R package Seurat and created a Seurat object for the dataset (NCBI GEO: GSE46980). By using the (NCBI GEO: GSE46980) Seurat object, we compared the main quality metrics used by Seurat, that is, the percentage of mitochondrial DNA, nCount_RNA, and nFeature_RNA, between typical, skewed, and dead cells (Figure 5F, typical cells identified by SkewC have high-quality values for the three measures compared with the skewed cells). Compared to skewed cells, typical cells have a higher level of nCount_RNA and nFeature_RNA, but a lower percentage of mitochondrial DNA genes.

To investigate the biological features, and relevance of the typical and skewed cells, we used the microscopic image created by the Fluidigm C1 chip, provided by the authors in (Islam et al., 2014), and computed the cell size of all cells (measured in pixels (Table S1). Figures 5G and 5H show the variation of the cell sizes in pixels, between typical and skewed cells. In Figure 5G, acceptable and unacceptable cells (Dataset authors’ annotation) are highlighted while the live and dead cells are highlighted in Figure 5H. Cell size analysis shows that typical and acceptable cells have a larger cell size compared to skewed and unacceptable cells.

### Effect of skewed cells on downstream analyses

**Figure 6:**
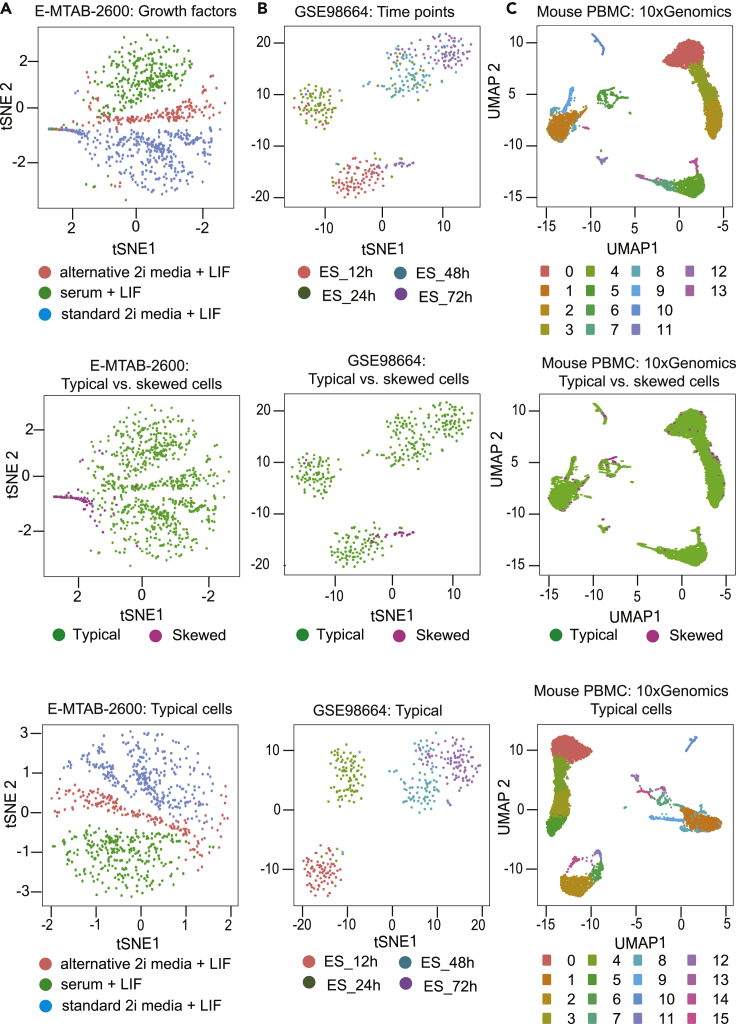
Effect of skewed cells on downstream analysis To evaluate the effect of the skewed cells when performing downstream analysis, *t*-SNE was generated for several datasets. (A) mESCs cells treated with three types of growth factors. On the top is the *t*-SNE before the classification and colored by the growth factor used, in the middle a *t*-SNE of the skewed and typical cells, on the bottom *t*-SNE after removing the skewed cells. (B) mESCs with four development time-points. Top *t*-SNE of the dataset before the classification. The cluster in the bottom shows the majority of the cells at 12 h, with few cells from 72 h. In the middle *t*-SNE we observed that the skewed cells are cells from 72 h. The bottom *t*-SNE shows better clustering of the cell per development time-point after filtering the skewed czells. (C) Change in the resulting (UMAP) after filtering the skewed cells.

As we noticed great variability in the mean expression of the HKGs between typical and skewed cells, we investigated the effect of skewed cells on the downstream analysis of scRNA-seq experiments. We analyzed the impact of filtering skewed cells on *t*-SNE using the mESCs dataset (Figures 6A and 6B). We also used the Uniform Manifold Approximation and Projection (UMAP) on the mouse PBMC from 10x Genomics (Figure 6C). *t*-SNE is a common dimensionality reduction technique used in scRNA-seq analysis and is usually performed after read count normalization (Hwang et al., 2018). In the first dataset (ArrayExpress: E-MTAB-2600) (Kim et al., 2015) generated by SMARTer protocol (Figure 6A), the top panel shows the *t*-SNE plot of all single cells, colored by the growth factors used in the experiment. It can be seen that some of the cells were misplaced into the wrong cluster (mis-clustering). The *t*-SNE plot (coloring based on the classification of typical and skewed cells) in the middle row, indicates that the majority of the skewed cells clustered together with few exceptions. The *t*-SNE plot in the bottom panel of (Figure 6A) shows the replotted *t*-SNE after filtering the skewed cells. The plotting of just the typical cells shows distinct clustering of the cells based on the growth factors, compared to the *t*-SNE before the filtering of the skewed cells. This shows the impact of the skewed cells on the clustering of the cells.

The next dataset (NCBI GEO: GSE98664) (Hayashi et al., 2018) is a time-course analysis of mESCs development generated by the RamDA-seq protocol (Figure 6B). The top panel illustrates *t*-SNE with four clusters (four time-points), each of which contains cells that do not belong to the same time-point (mis-clustered). The 24 and 48 h cells were intermixed with skewed cells in *t*-SNE. The mis-clustered cells were in fact skewed cells; these might be stopped in their development process, but, were mis-annotated as developing cells. The middle *t*-SNE plot, colored by typical and skewed cells, shows the appearance of skewed cells in two clusters (ES_12h & ES_24h). The bottom *t*-SNE illustrates re-clustering of the dataset after filtering the skewed cells. The plot shows a clear improvement in the clustering result. In the final *t*-SNE, after filtering of the skewed cells, the set of cells in the time-point ES_72h from the ES_12h cluster was removed and the ES_24h skewed cells from the ES_48h cluster were removed. Removing the skewed cells (mainly from 12 h) distinctly separated ES_24h and ES_48h cells. To identify the marker genes that were contributing to the change in the *t*-SNE after removing the skewed cells, we independently performed UMAP clustering and FindMarkers function from Seurat (Butler et al., 2018). We identified a set of marker genes contributing to the mis-classification of the ES_12h cells to ES_24h single cells. This indicated that SkewC filtered (skewed) single cells with noisy genes. The analysis results and the UMAP plots are available at: http://single-cell.riken.jp/SkewC_Figs_DataFiles/MainFigure_6/.

In the third dataset (Mouse PBMC: 10xGenomics), generated by the 10x Genomics chromium protocol (Figure 6C), the skewed cells cluster appeared in different clusters on a UMAP plot (top of Figure 6C) (notice number of cluster = 14). After filtering the skewed cells, UMAP showed more distinct clusters (n = 17) consisting of only typical cells.

All of the above examples demonstrate the strong impact of skewed cells on the downstream clustering results. Therefore, the identification and filtering of the skewed cells are important to consider in any downstream analyses.

### Enhancing the performance of the gene body coverage computation for the scRNA-seq protocol

**Figure 7:**
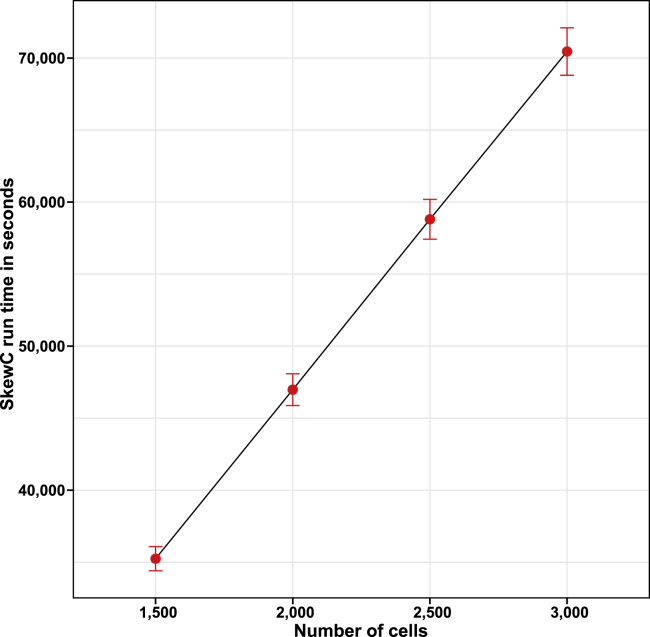
Performance evaluation of SkewC The figure demonstrates the scalability of SkewC and showing linear association between SkewC run time (y axis) and the number of cells (x axis). The error bars represents the standard error of the mean (SEM).

We implemented a tool that is capable of computing the gene body coverage of more than 15,000 cells generated by any scRNA-seq protocol. The tool integrated into SkewC to compute the gene body coverage, shown in Figures 1 and 3. SkewC accepts only BAM files compared to other tools that required indexed BAM files. Our pipeline is capable of handling barcoded BAM, generated by the 10x Genomics Cell Ranger pipeline (https://www.10xgenomics.com/). To our knowledge, this is the first tool designed to use gene body coverages for the cellular-level quality assessment with scRNA-seq datasets. The results from the performance and scalability analysis are shown in Figure 7. The analysis shows a linear association between the number of cells and SkewC run time. The performance evaluation result shown in Figure 7 was conducted using only 10 cores, the user will be able to drastically reduce the computation time by increasing the number of cores.

### Incorporating SkewC in the scRNA-seq analysis workflow

**Figure 8:**
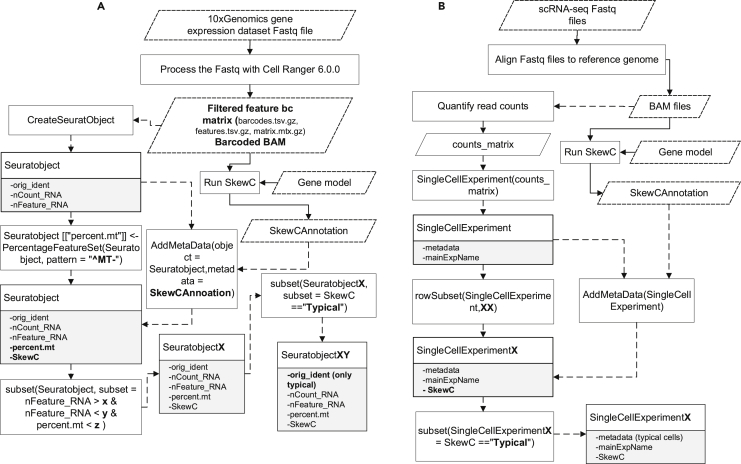
Incorporating SkewC with scRNA-seq workflow (A) Incorporating SkewC in two types of scRNA-seq analysis workflows. Dashed lined indicates process run in R project continues lines represented process that run outside R project (A) analysis of 10xGenomics gene expression dataset. The output from Cell Ranger used as input for SkewC and Surat R package. SkewC annotation added as metadata column to Seurat object. (B) The second type of scRNA-seq analysis for non 10xGenomics dataset using the SingleCellExperiment class.

Figure 2 shows the implementation of SkewC, and the final results were a list of annotated cell ids work barcodes each cell in the dataset will either be annotated as typical or skewed. SkewC generates the annotation result in two different formats, the first as two text files named as (TypicalCellsID.tsv and SkewedCellsID.tsv), the second as R data frame object SkewCAnnoation.rds. The data frame SkewCAnnoation consists of two columns the cell_id and the SkewC. To incorporate SkewC result in the scRNA-seq analysis workflow, we suggested the workflow in (Figure 8). Figure 8A consider the 10xGenomics gene expression dataset, this workflow integrates result from Cell Ranger, Seurat (Butler et al., 2018) and SkewC. Although (Figure 8B) consider non 10xGenomics scRNA-seq dataset to build R SingleCellExperiment (Amezquita et al., 2020) class.

## Discussion

Advances in scRNA-seq have already had an impact on biology and medical science and will continue to do so. It has enabled the investigation of transcriptomic variation between individual cells, thereby enabling the discovery of new cell types; allowed for analysis of cellular response to stimulation; also analysis of the nature and dynamics of cell differentiation and reprogramming; and the study of transcriptional stochasticity (Sandberg, 2014). Despite these technical advances, several challenges still remain and must be understood for improved interpretation of available and future data. There is high variability in the performance of scRNA-seq protocols in terms of coverage, accuracy, and specificity, impacting the quality of data generated by different protocols (Ziegenhain et al., 2017; Svensson et al., 2017). The variability among scRNA-seq protocols and the quality of the datasets produced might also impact global efforts to map transcription in human and mouse cells (Regev et al., 2017; Consortium et al., 2018).

Our analysis identified wide differences in patterns of gene coverage generated from the same cell types by different scRNA-seq protocols. Based on the analysis of gene body coverage, we identified two classes of cells in any dataset: cells with method-specific (prototypical) distribution pattern of gene body coverage (typical), and cells with a skewed distribution pattern (skewed). Each of the scRNA-seq protocols yields different gene body coverage so the pattern of a typical distribution will vary among datasets. A skewed distribution, with excess 3′ end bias observed, could be attributed to a technical or biological error during any of the experimental steps; this could be reaction failure or cell death thus triggering mRNA degradation. During embryo development, skewed distribution coverage suggested the induction of maternal transcript degradation at early genome activation in the earliest developmental stages (La Manno et al., 2018), as maternal transcript degradation takes place after fertilization until day 3 (Dobson et al., 2004; Töhönen et al., 2015). The skewed live cells might potentially be quiescent satellite cells, a form of (**G**_0_ cell-cycle phase), cells enter **G**_0_ phase (resting) owing to varying environmental factors. The common feature between the identified skewed cells and the quiescent satellite cells is the low RNA content (Hüttmann et al., 2001; Fukada et al., 2007).

SkewC is based on the observation of the two distribution patterns in the gene body coverage of scRNA-seq protocols and the identification of typical and skewed cells. The skewed cells might result from a technical failure of the reaction, cell death, or a biological process, for example, related to embryo development. Another biological characteristic of the skewed live cells was the low content of RNA and RNA degradation. This could be owing to biological processes, for example, by miRNA-mediated post-transcriptional regulation. These features of the skewed cells were observed in quiescent stem cells (reviewed in (Cheung and Rando, 2013)), although we have not confirmed the biological background in the present study.

We can see the impact of gene body coverage skewness in many aspects. In the expression of housekeeping genes, we identified significant differences between typical and skewed cells. Regarding the cell size and cell cycle of typical and skewed cells, typical cells show a strong correlation between cell size and cell-cycle phase; this is in contrast to skewed cells. The differential gene expression analysis demonstrated that skewed cells showed different expression profiles of the top expressed genes, compared to typical cells. We further assessed the impact of filtering out the skewed cells from downstream analyses (clustering and differential gene expression analysis) and found that the exclusion of skewed cells changed their clustering results. Mis-clustering may lead to false discovery resulting from skewed cells. From these results, we recommend that skewed cells should be identified and excluded from any downstream analyses during scRNA-seq experiments.

In conclusion, our results demonstrated that the presence of skewed cells influences the data analysis and interpretation of scRNA-seq data and therefore, a QC method such as SkewC, used to segregate typical cells and skewed cells in these datasets, should be standard procedure in any scRNA-seq experiment. Indeed, the SkewC method described here can be easily integrated into any scRNA-seq data analysis workflow.

### Limitations of the study

Our investigation of gene body coverage yields by scRNA-seq protocols lead to the identification of two classes of scRNA-seq cells based on their gene body coverage skewness; these classes are typical and skewed cells. However, the exact cause of the skewed cells could be attributed to technical failure or human error during experimental procedures, failure of scRNA-seq protocol, and/or owing to biological characteristics of these so-called skewed cells. To have a better understanding of the biological characteristics of skewed cells, further efforts and investigation are required.

## STAR★Methods

### Key resources table

**Table:** 

REAGENT or RESOURCE	SOURCE	IDENTIFIER
**Deposited data**		

GSE143607	New dataset	https://www.ncbi.nlm.nih.gov/geo/query/acc.cgi?acc=GSE143607
E-MTAB-2600	ArrayExpress	https://www.ebi.ac.uk/arrayexpress/experiments/E-MTAB-2600/
E-MTAB-2805	ArrayExpress	https://www.ebi.ac.uk/arrayexpress/experiments/E-MTAB-2805/
E-MTAB-2512	ArrayExpress	https://www.ebi.ac.uk/arrayexpress/experiments/E-MTAB-2512
E-MTAB-3857	ArrayExpress	https://www.ebi.ac.uk/arrayexpress/experiments/E-MTAB-3857
E-MTAB-4619	ArrayExpress	https://www.ebi.ac.uk/arrayexpress/experiments/E-MTAB-4619
E-MTAB-3929	ArrayExpress	https://www.ebi.ac.uk/arrayexpress/experiments/E-MTAB-3929
PBMCs from a Healthy Donor: Whole Transcriptome Analysis (v3.1 chemisty)	10xGenomics	https://support.10xgenomics.com/single-cell-gene-expression/datasets
Hodgkin's Lymphoma, Dissociated Tumor: Whole Transcriptome Analysis (v3.1 chemisty)	10xGenomics	https://support.10xgenomics.com/single-cell-gene-expression/datasets
1k Brain Nuclei from an E18 Mouse (v2 chemistry)	10xGenomics	https://support.10xgenomics.com/single-cell-gene-expression/datasets
Pan T Cells isolated from mononuclear cells of a healthy donor	10xGenomics	https://www.10xgenomics.com/resources/datasets/4-k-pan-t-cells-from-a-healthy-donor-2-standard-2-1-0
1k PBMCs from a Healthy Donor (v3 chemistry)	10xGenomics	https://support.10xgenomics.com/single-cell-gene-expression/datasets
PRJDB5282	DDBJ	https://ddbj.nig.ac.jp/BPSearch/bioproject?acc=PRJDB5282
PRJEB8994	ENA	https://www.ebi.ac.uk/ena/browser/view/PRJEB8994
GSE42268	NCBI GEO	https://www.ncbi.nlm.nih.gov/geo/query/acc.cgi?acc=GSE42268
GSE53386	NCBI GEO	https://www.ncbi.nlm.nih.gov/geo/query/acc.cgi?acc=GSE53386
GSE98664	NCBI GEO	https://www.ncbi.nlm.nih.gov/geo/query/acc.cgi?acc=GSE98664
GSE74833	NCBI GEO	https://www.ncbi.nlm.nih.gov/geo/query/acc.cgi?acc=GSE74833
GSE67310	NCBI GEO	https://www.ncbi.nlm.nih.gov/geo/query/acc.cgi?acc=GSE67310
GSE45719	NCBI GEO	https://www.ncbi.nlm.nih.gov/geo/query/acc.cgi?acc=GSE45719
GSE75659	NCBI GEO	https://www.ncbi.nlm.nih.gov/geo/query/acc.cgi?acc=GSE75659
GSE59114	NCBI GEO	https://www.ncbi.nlm.nih.gov/geo/query/acc.cgi?acc=GSE59114
GSE68981	NCBI GEO	https://www.ncbi.nlm.nih.gov/geo/query/acc.cgi?acc=GSE68981
GSE70657	NCBI GEO	https://www.ncbi.nlm.nih.gov/geo/query/acc.cgi?acc=GSE70657
GSE56638	NCBI GEO	https://www.ncbi.nlm.nih.gov/geo/query/acc.cgi?acc=GSE56638
GSE64016	NCBI GEO	https://www.ncbi.nlm.nih.gov/geo/query/acc.cgi?acc=GSE64016
GSE75748	NCBI GEO	https://www.ncbi.nlm.nih.gov/geo/query/acc.cgi?acc=GSE75748
GSE70151	NCBI GEO	https://www.ncbi.nlm.nih.gov/geo/query/acc.cgi?acc=GSE70151
GSE29087	NCBI GEO	https://www.ncbi.nlm.nih.gov/geo/query/acc.cgi?acc=GSE29087
GSE46980	NCBI GEO	https://www.ncbi.nlm.nih.gov/geo/query/acc.cgi?acc=GSE46980
GSE54695	NCBI GEO	https://www.ncbi.nlm.nih.gov/geo/query/acc.cgi?acc=GSE54695
GSE78779	NCBI GEO	https://www.ncbi.nlm.nih.gov/geo/query/acc.cgi?acc=GSE78779

**Experimental models: cell lines**		

MCF10A	ATCC	CRL-10317

**Software and algorithms**		

NCBI Entrez Programming Utilities	NCBI	https://www.ncbi.nlm.nih.gov/books/NBK25501/
Bioconductor	(Gentleman et al., 2004)	https://www.bioconductor.org/
DESeq2	(Love et al., 2014)	http://bioconductor.org/packages/release/http://bioconductor.org/packages/release/bioc/html/DESeq2.html
Python	Python programming language	https://www.python.org/
ImageJ	NIH	https://imagej.nih.gov/ij/
Seurat	https://doi.org/10.1016/j.cell.2019.05.031	https://satijalab.org/seurat/

### Resource availability

#### Lead contact

Further information and requests for source code and data processing protocols should be directed to and will be fulfilled by the Lead contact Takeya KASUKAWA (takeya.kasukawa@riken.jp)

#### Materials availability

This study did not generate new unique reagents. The raw reads of all used datasets are available from INSDC sites. The data are also available in SCPortalen, a single-cell database in which we deposited all results from this study at (SCPortalen: http://single-cell.riken.jp/).

### Method details

#### Study design

Based on the objective of the scRNA-seq experiment, the protocols are divided in two categories, full-length sequence profiling or transcript end-tagging (5′ or 3′). In full-length sequence protocols (SMARTer, Smart-Seq, SUPer-Seq, RamDA-seq, etc.), the sequence reads cover the entire gene body (5′ to 3′ end) and are potentially able to quantify gene and transcript isoforms. The end-tagging based sequencing protocols (C1 CAGE, CEL-Seq, CEL-Seq2, STRT, 10x Chromium Single Cell 3' end etc.), target one end of the transcript (5′ or 3' end) and are used to identify promoters (5′ tagging) or give an estimate of transcript abundance. We compared and analyzed scRNA-seq protocols using approaches different from published studies (Ziegenhain et al., 2017; Svensson et al., 2017). In particular, we evaluated the capability and power of each protocol in terms of the full-length transcript coverage. We analyzed 33 datasets, produced with 15 different protocols, representing commonly used scRNA-seq methods of the above two categories. Based on the target read capture strategy of the scRNA-seq method, five datasets measured gene expression at the 5′-end of the transcript (STRT, C1 CAGE and 10x Chromium 5′-end), 20 datasets measured gene expression of the full-length transcript (SMARTer, Smart-Seq, RamDA-seq, SUPer-Seq, Quartz-Seq, C1 single-cell mRNA-Seq, Smart-Seq2 and TruSeq), and eight studies measured gene expression at the 3′-end of the transcript (CEL-Seq, CEL-Seq2, Drop-Seq and 10x Chromium 3′-end).

#### Study dataset

In this study, we reanalyzed published human and mouse scRNA-seq datasets acquired from International Nucleotide Sequence Database Collaboration (INSDC) and the 10x Genomics support site. We also used a 10x Genomics dataset produced by ourselves. To perform fair comparison among different protocols and to make the results comparable, we categorized the analysis datasets based on the organism and cell type (Tables S2 and S3).

#### International Nucleotide Sequence Database Collaboration (INSDC) dataset

The method for collecting and processing raw reads of scRNA-seq from public databases were illustrated in our previous report (Abugessaisa et al., 2018) and listed in Tables S2 and S3 (Kim et al., 2015; Buettner et al., 2015; Sasagawa et al., 2013; Fan et al., 2015; Hayashi et al., 2018; Mahata et al., 2014; Stubbington et al., 2016; Proserpio et al., 2016; Zhang et al., 2019; Treutlein et al., 2016; Deng et al., 2014; Reinius et al., 2016; Kowalczyk et al., 2015; Yang et al., 2017; Grover et al., 2016; Dueck et al., 2015; Leng et al., 2015; Petropoulos et al., 2016; Chu et al., 2016; Macosko et al., 2015; Islam et al., 2011, 2014; Kouno et al., 2019; Töhönen et al., 2015; Grün et al., 2014; Hashimshony et al., 2016). In brief terms, published scRNA-seq data were collected by searching PubMed for human and mouse scRNA-seq articles. Our aim was to include different types of cells generated by different technology platforms. We retrieved study accession number(s) of the original data deposited to INSDC. The study accession numbers were used to retrieve sequence read files and metadata files from INSDC sites (DDBJ, EMBL-EBI, NCBI). To obtain FASTQ files, we implemented an automated program using the NCBI SRA Toolkit (Leinonen et al., 2010). Metadata about each dataset were collected as well. This metadata contains information about the cell type, protocol, sequence platform, cell isolation techniques, etc. We implemented an automated script to retrieve dataset metadata utilizing the Entrez Programming Utilities (E-utilities) from NCBI (NCBI Resource Coordinators, 2017). The whole scripts are available at https://github.com/LSBDT/SCPortalen.

#### 10x genomics public datasets

We downloaded six single cell gene expression datasets from 10x Genomics portal (https://support.10xgenomics.com/single-cell-gene-expression/datasets). The datasets are human PBMCs of a healthy donor: 5' end gene expression and cell surface protein (v1.0 chemistry) (8,258 cells); mouse PBMCs from C57BL/6 mice: 5′ gene expression (v1.0 chemistry) (8,500 cells); human PBMCs from a healthy donor: whole transcriptome analysis single cell 3′ gene expression v3.1 dual index library (10,194 cells); human Hodgkin's lymphoma, dissociated tumor: whole transcriptome analysis, single cell 3′ gene expression v3.1 dual index library (3,394 cells); human 1k PBMCs from a healthy donor - gene expression and cell surface protein v3 chemistry (714 cells); and 1k brain nuclei from an E18 mouse v2 chemistry (954 cells).

#### Production of 10x Genomics Chromium dataset of human MCF10A cells

We generated our own 10x Genomic Chromium dataset from MCF10A cells (ATCC). The cells were grown in DMEM/F12(1:1) as described in a previous report (Watanabe et al., 2019). RNA-seq libraries were prepared using 10x Chromium Single Cell 3'–end Reagent Kits (v2 Chemistry), following manufacturer’s instructions. The libraries were sequenced using paired-end sequencing (26 bp Read 1 and 98 bp Read 2) with a single sample index (8 bp) on an Illumina HiSeq 2500. Raw reads and processed data are deposited in NCBI GEO (NCBI GEO: GSE143607).

#### Data processing of the raw sequence reads

For the raw sequence data downloaded from INSDC, we ran a basic QC procedure to obtain quality assessment metrics of the raw sequence reads. The QC procedure included testing of all FASTQ files with the FastQC tool (http://www.bioinformatics.babraham.ac.uk/projects/fastqc/) to identify any quality issues. Additional QC of the raw reads included the count of raw tags. The raw sequence reads were aligned to recent reference genome builds [GRCh38 (human) or GRCm38 (mouse) genome assembly]. We used STAR software (version 2.5.1b) (Dobin et al., 2013) with default settings and GENCODE gene annotations (v24 for human and vM9 for mouse) for all datasets apart from one dataset (NCBI GEO: GSE98664) in which we used GENCODE vM22. Aligned reads, in BAM file format, together with the log files generated by STAR, were used to obtain quality assessment metrics including: total read count, number of uniquely mapped reads and assigned reads (mapped reads assigned to gene). The mapping ratio, counts of mapped reads, unmapped and multi-mapped reads were summarized using SAMtools software (Li et al., 2009). For the MCF10A 10x Genomic Chromium dataset, we used Cell Ranger version 2.1.1 for data processing. The datasets from the 10x Genomics web site were processed with different versions of Cell Ranger.

#### Read count summarization and expression normalization

We quantified gene expression counts using featureCounts (in the Subread package Version 1.5.0-p1) (Liao et al., 2014) to obtain expression matrices. The gene expression counts were normalized into transcripts per million (TPM) and fragments per kilobase of transcript per million mapped reads (FPKM), for datasets generated by full length scRNA-seq protocol, and ag per million (TPM) for dataset generated by end tagging protocols. We produced a single gene expression table for each study. For the 10x Genomics datasets, we used the computed feature-barcode matrices in the MEX format provided by the Cell Ranger software.

#### Computation and visualization of gene body coverage

We implemented a fast pipeline that imitated RSeQC (Wang et al., 2012) to compute the gene body coverage for scRNA-seq datasets. The original program has been used to check if read coverage is uniform and if there is any 5′ or 3′ end bias. The input for the program requires BAM files or Barcoded BAM files, in case of 10x Genomics data, and a gene model in the BED format. Human and mouse gene models were downloaded from http://rseqc.sourceforge.net/#download-gene-models-update-on-08-07-2014. The gene model BED files for human hg38_Gencode_V28.bed.gz and for mouse mm10_Gencode_VM18.bed.gz were preprocessed to filter ribosomal RNA and transfer RNA.

The result of the program is a vector of values consisting of normalized coverages within its gene body from the 5′-end to the 3′-end, bound to 0–100 (positions). The value for each position ranges from 0–1, where 0 indicates no coverage and 1 indicates full coverage at the position on the gene body. The vector of the normalized values was post-processed in several steps for visualization and to study the normality and skewness of the coverage (see Figure 1 for the procedure).

#### SkewC implementation

The overall implementation and workflow of SkewC is illustrated in Figure 2. The method begins with computation of the gene body coverage and then applies several post-processing steps to the resulting gene body coverage matrix to obtain the mean coverage. We used trim clustering (R function tclust (Fritz et al., 2012)). Each observation inputted to tclust is either assigned to a cluster or trimmed. tclust function is used to cluster the coverage matrix, whose inputs are the number of the clusters (K = 1) and Alpha (α) value. The α-value ranges between 0-1 and varies between datasets, therefore, SkewC auto-approximates the α using the trimmed likelihood to select the optimum α values. In addition to auto-approximation of α, SkewC enables the user to manually select α; an example of the output of SkewC with manual selection of α values for the data PBMC_8K from 10x Genomics can be found in Online supplementary figures: http://single-cell.riken.jp/SkewC_Online_Suppl_Figs/PBMC_8K_ManualAlpha/. And for auto approximate α value for the same dataset in Online supplementary figures http://single-cell.riken.jp/SkewC_Online_Suppl_Figs/PBMC_8K_AutoAlpha/. As demonstrated in the figure, change of α values will change the clustering results (Figure S8). Since scRNA-seq experiments profile thousands of cells, we considered the computational cost and runtime of SkewC when the number of profiled cells increases. To measure the scalability of SkewC, we analyzed the dataset ‘pbmc_8k’ from 10x Genomics 3′ end gene expression (total number of cells n = 8,000). We split 10xGenomics post-processed BAM and the ‘barcodes.tsv’ into 6 files (dataset). This result in 6 post-sorted BAM files and 6 ‘barcodes.tsv’ files. We run each of the 6 dataset four times using 10 cores and measure the time in seconds.

#### Cell size estimation of the mESC dataset ( NCBI GEO: GSE46980)

The dataset (NCBI GEO: GSE46980) of mESCs (Islam et al., 2014) was generated by the STRT protocol and provided full annotation of the quality status of each cell (n = 96). The authors classified each cell as either dead (depleted before cell capture by the flow-cell) or live. The live cells were further classified as either low or good quality cells (see the original paper (Islam et al., 2014) for details on how the annotation was performed). We used this dataset to compare our QC method of typical and skewed cells. Additionally, the dataset provided a microscopic image of the Fluidigm C1 chip. In the microscopic image, each Fluidigm C1 chip (a 96-well plate) was imaged after cell capture and a grid of thumbnails was generated for each chip. To verify some of the morphological phenotypes of the typical and skewed cells, we estimated morphological properties of the cells based on the microscopic image, cell size, areas, circularity, skewness roundness and solidity; these were calculated using the ImageJ tool (Schneider et al., 2012).

### Quantification and statistical analysis

Unless otherwise indicated, all p-values were obtained with wilcox.test and alternative = "greater". We used “Hochberg” method for adjusted P-value. multiple correction method (Table S4). In all boxplots, center lines indicate median values and box heights indicate the inter-quartile range of data. The ggplot2 library from R software version 3.6.1 (2019-07-05) was used for plotting all plots and figures. Adobe Illustrator 1.0 was used for formatting all figures.

## Data Availability

1. SkewC Docker container image can be download from our GitHub https://github.com/LSBDT/SkewC 2. R Markdowns used to process datasets from INSDC are available from our GitHub page: https://github.com/LSBDT/SCPortalen. 3. The dataset used to generate all figures for this manuscript are under http://single-cell.riken.jp/SkewC_Figs_DataFiles/ 4. The R script files used to generate all figures for this manuscript can be download from GitHub https://github.com/LSBDT/SkewC_Manuscript 5. Additional figures that cannot be embedded to the report are available from (SCPortalen: http://single-cell.riken.jp/SkewC_Online_Suppl_Figs/) 6. Computed gene body coverage R files for all datasets are available for download from (SCPortalen: http://single-cell.riken.jp/Database_Files/gene_body_coverage/) 7. The accession numbers for the 10x Genomics dataset generated in this manuscript is (NCBI GEO: GSE143607) in NCBI GEO (gene expression omnibus). 8. Any additional information required to reanalyze the data reported in this paper is available from the lead contact upon request.

## Supplemental information

Document S1. Figures S1–S9

Table S1. Analysis of the microscopic image for the dataset GSE46982, related to Figure 5G-H

Table S2. Sample design and tested single-cell RNA sequence protocols, related to STAR Methods

Table S3. List of the datasets used in the analysis, related to STAR Methods

Table S4. P-value and adjusted p-value for main, Figure 4, related to STAR Methods
