# Supplementary material for: SkewC: Identifying cells with skewed gene body coverage in single-cell RNA sequencing data
Source: iScience. 2022 Jan 15;25(2):103777. doi: 10.1016/j.isci.2022.103777 (PMC8819117; doi:10.1016/j.isci.2022.103777)
Supplement: Document S1. Figures S1–S9 [file mmc1.pdf]

iScience, Volume *25*

Supplemental information

SkewC: Identifying cells with skewed gene body coverage in single-cell RNA sequencing data

Imad Abugessaisa, Akira Hasegawa, Shuhei Noguchi, Melissa Cardon, Kazuhide Watanabe, Masataka Takahashi, Harukazu Suzuki, Shintaro Katayama, Juha Kere, and Takeya Kasukawa

Figure S1. Gene coverage skewness and variation in expression among single-cell RNA-Seq protocols using mouse CD4 T cells, related to Figure 1.

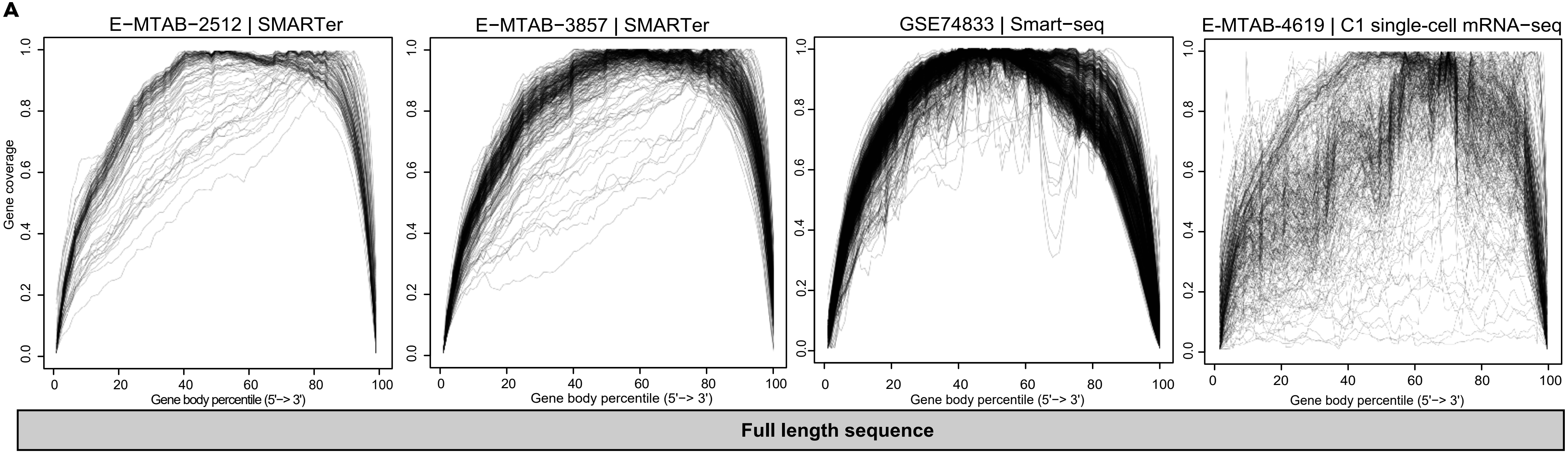

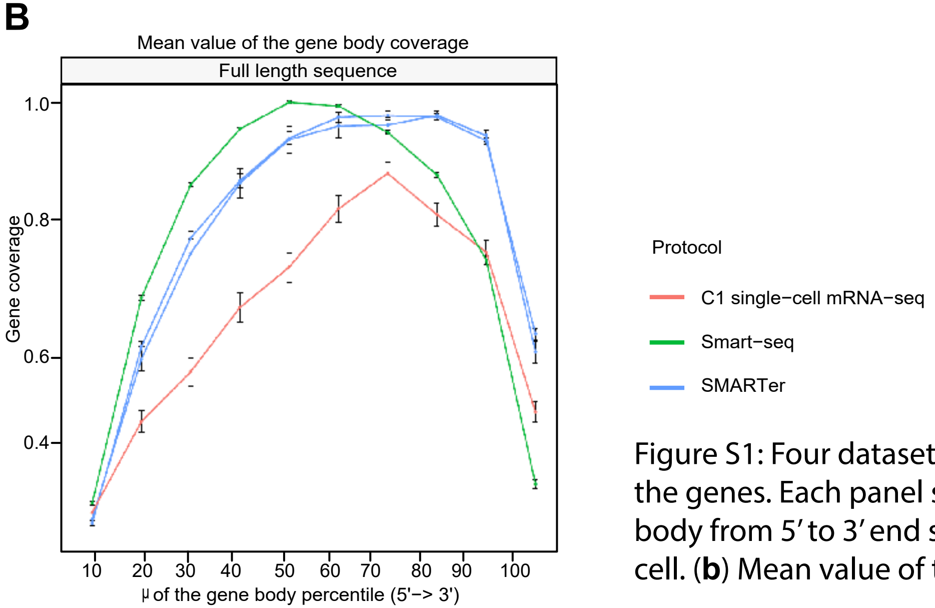

Figure S1: Four datasets from mouse CD4 T cells. (**a**) Distribution of the mapped reads (tags) across the genes. Each panel shows gene body coverage percentile per dataset. The x-axis represents the gene body from 5′ to 3′ end scaled from 0-100, and the y-axis gene coverage (0-1). Each line represents a single cell. (**b**) Mean value of the gene body coverage.

Figure S2. Gene coverage skewness and variation in expression among single-cell RNA-Seq protocols using mouse fibroblast cells, related to Figure 1.

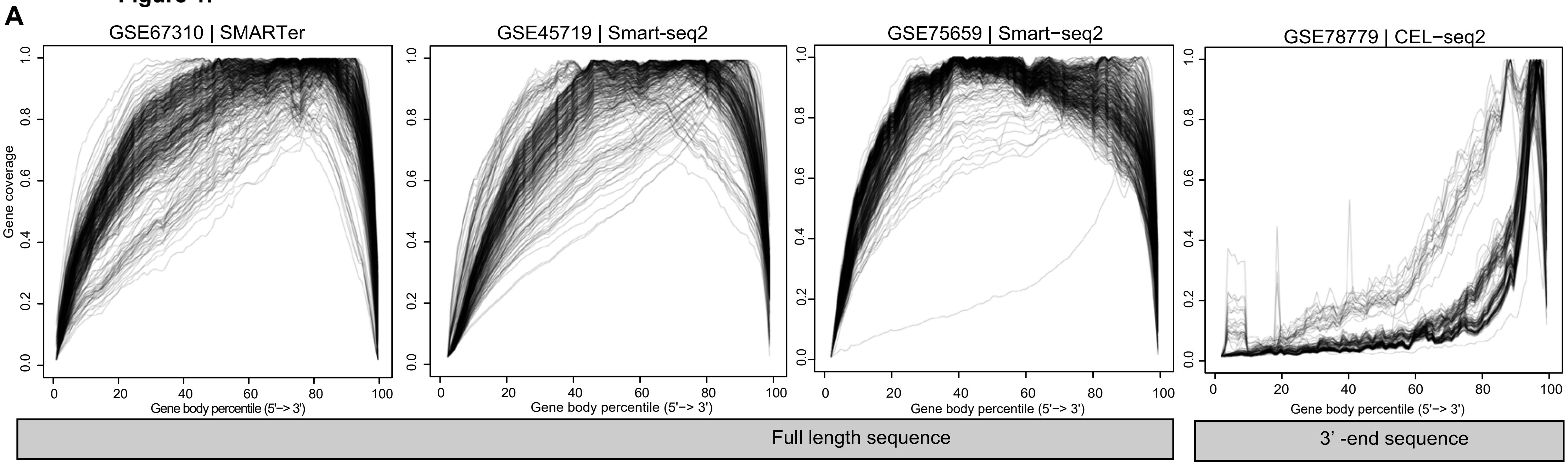

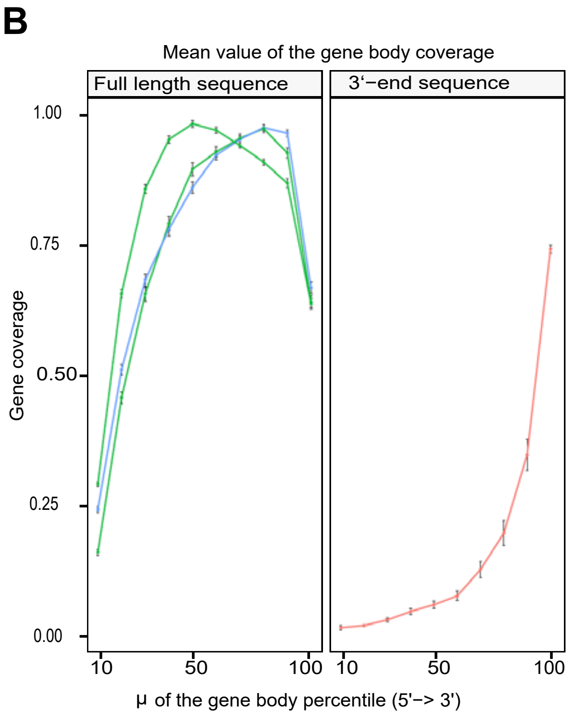

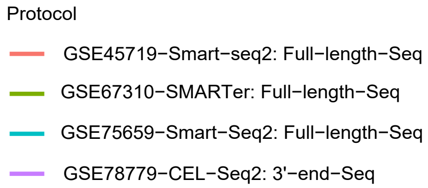

Figure S2 Four datasets from mouse fibroblast. (**a**) Distribution of the mapped reads (tags) across the genes. Each panel shows gene body coverage percentile per dataset. The x-axis represents the gene body from 5′ to 3′ end scaled from 0-100, and the y-axis gene coverage (0-1). Each line represents a single cell. (**b**) Mean value of the gene body coverage.

Figure S3. Gene coverage skewness and variation in expression among single-cell RNA-Seq protocols using mouse hematopoietic stem cells (HSCs), related to Figure 1.

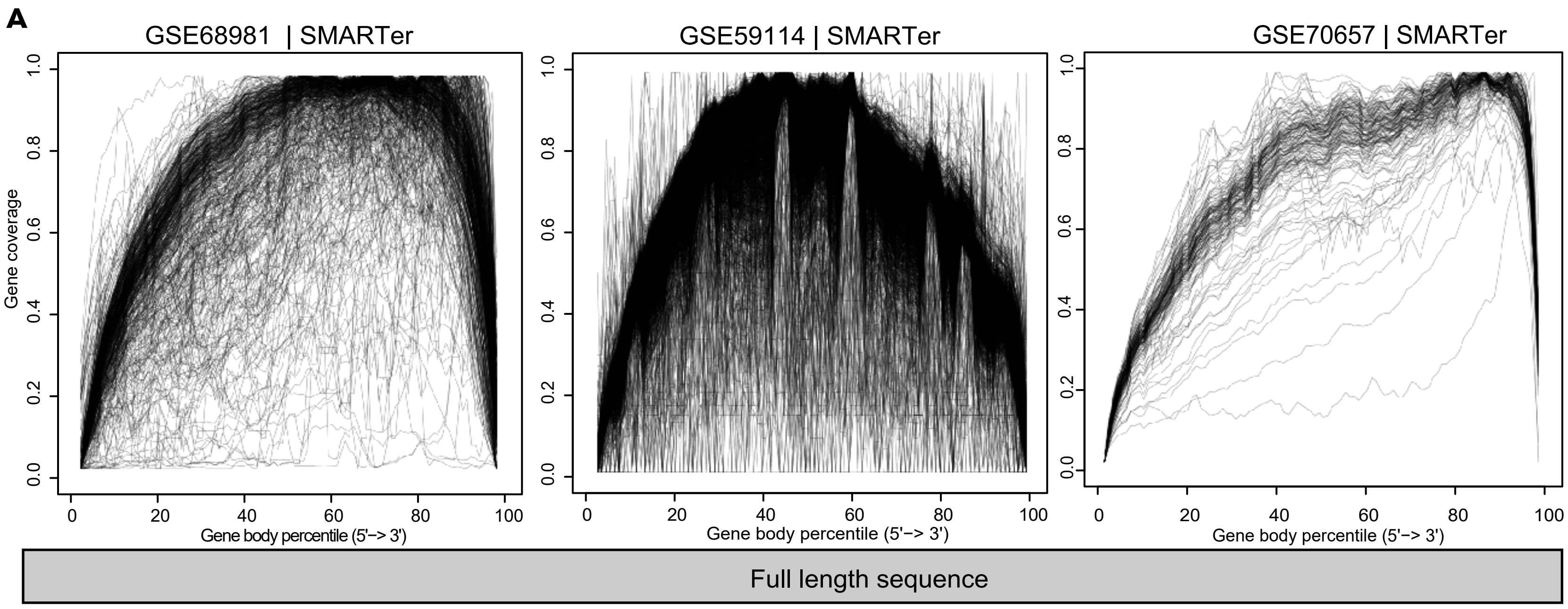

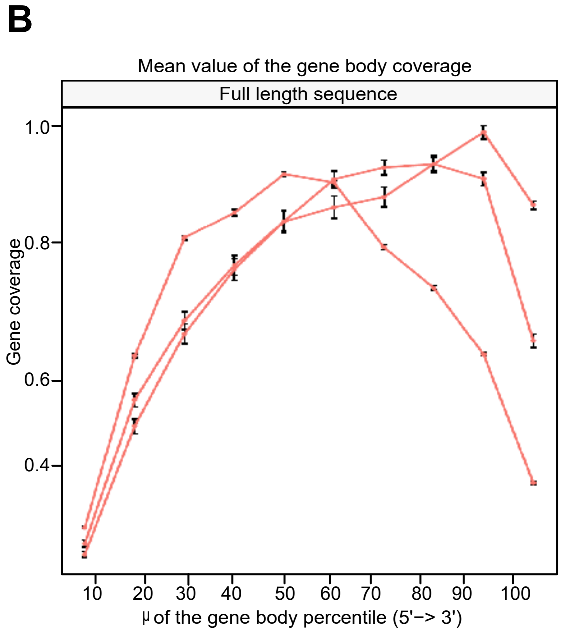

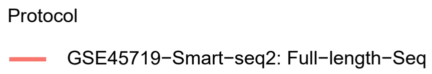

Figure S3: Three datasets from mouse hematopoietic stem cells (HSCs). (**a**) Distribution of the mapped reads (tags) across the genes. Each panel shows gene body coverage percentile per dataset. The x-axis represents the gene body from 5′ to 3′ end scaled from 0-100, and the y-axis gene coverage (0-1). Each line represents a single cell. (**b**) Mean calculated for bin size = 10.

Figure S4. Gene coverage skewness and variation in expression among single-cell RNA-Seq protocols using human embryonic stem cells (hESCs), related to Figure 1.

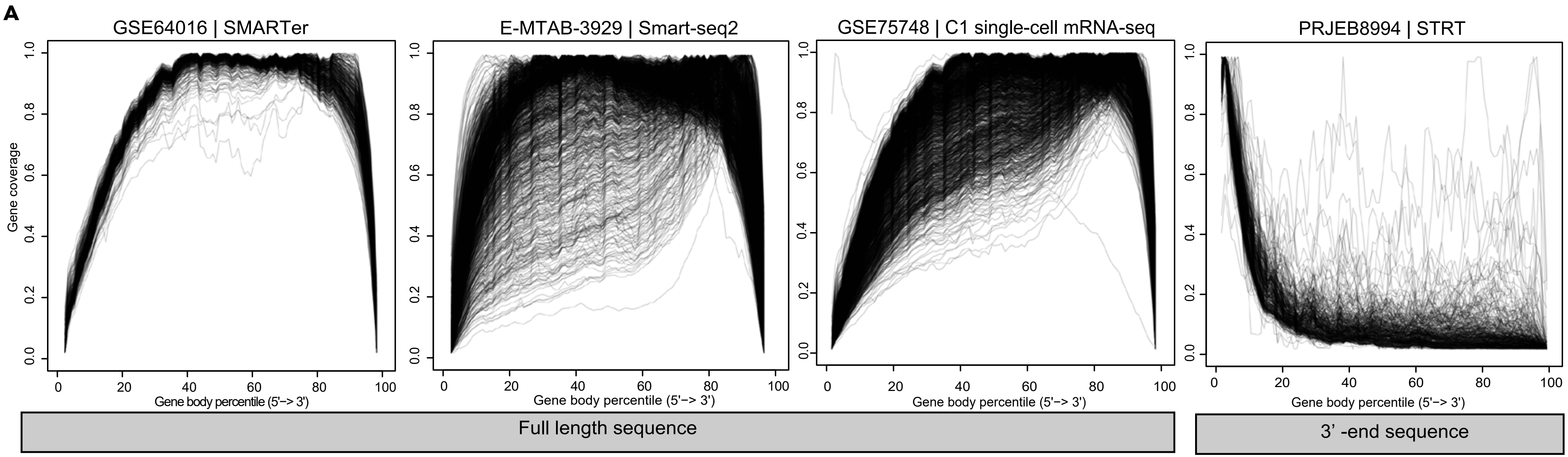

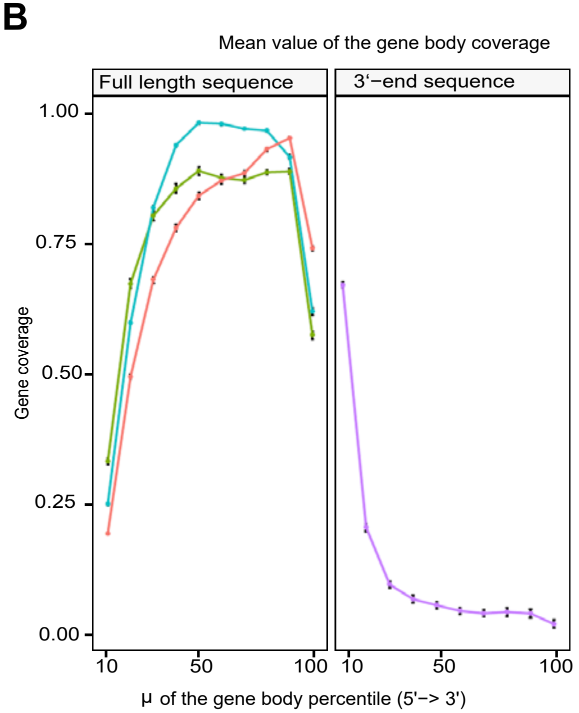

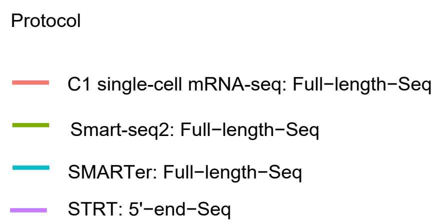

Figure S4: Four datasets from mouse human embryo cells. (**a**) Distribution of the mapped reads (tags) across the genes. Each panel shows gene body coverage percentile per dataset. The x-axis represents the gene body from 5' to 3' end scaled from 0-100, and the y-axis gene coverage (0-1). Each line represents a single cell. (**b**) Mean calculated for bin size = 10.

Figure S5. Gene coverage skewness and variation in expression among sc-RNA-Seq protocols using mouse adipocyte and mouse PBMC cells, related to Figure 1.

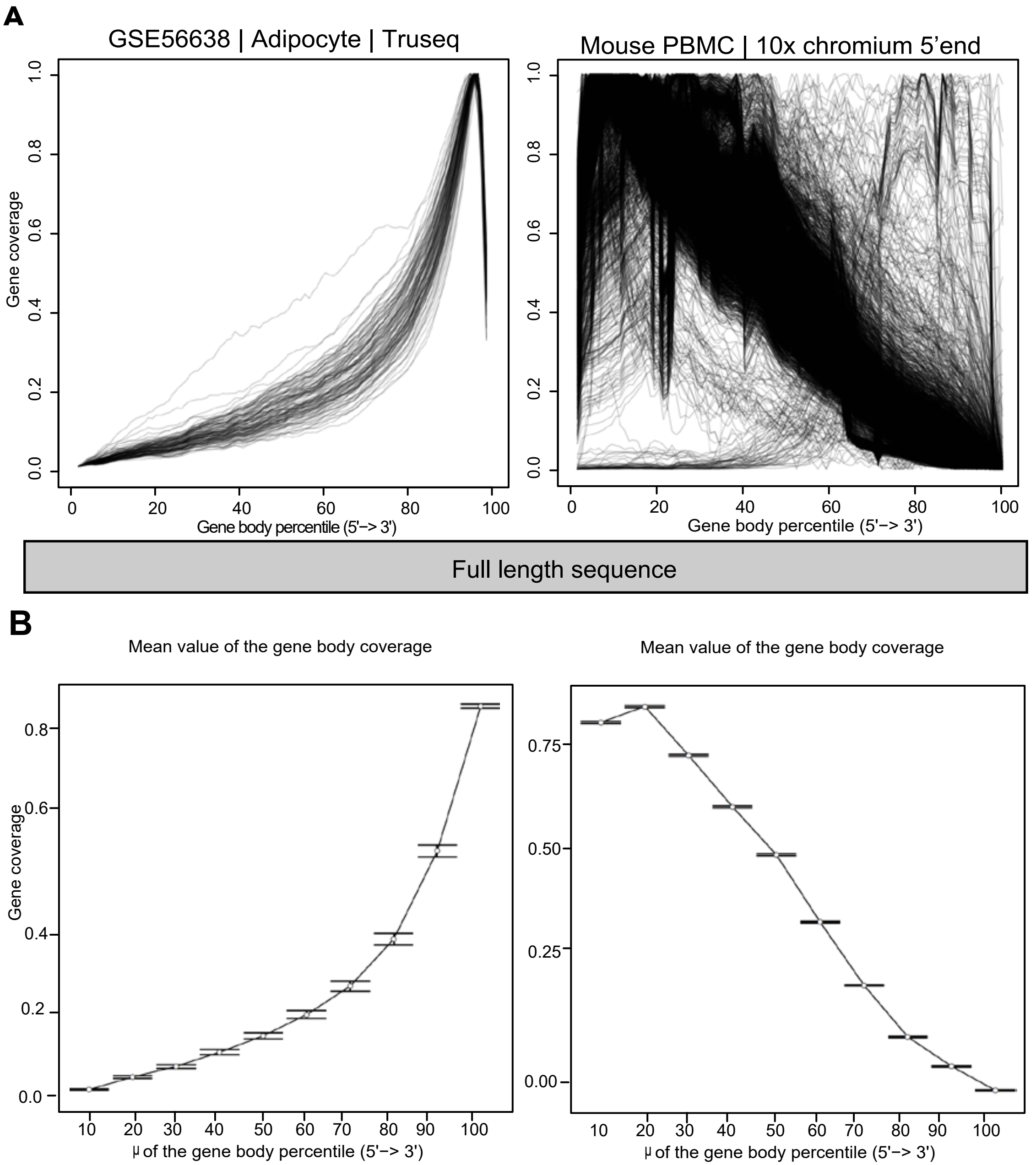

Figure S5: Two mouse dataset, the GSE56638 from Adipocyte tissue generated by Truseq and PBMC generated by 10x chromium 5' end. (**a**) Distribution of the mapped reads (tags) across the genes. Each panel shows gene body coverage percentile per dataset. The x-axis represents the gene body from 5' to 3' end scaled from 0-100, and the y-axis gene coverage (0-1). Each line represents a single cell. (**b**) Mean calculated for bin size = 10.

Figure S6. Gene coverage skewness and variation in expression among single-cell RNA-Seq protocols using different human cell types, related to Figure 1.

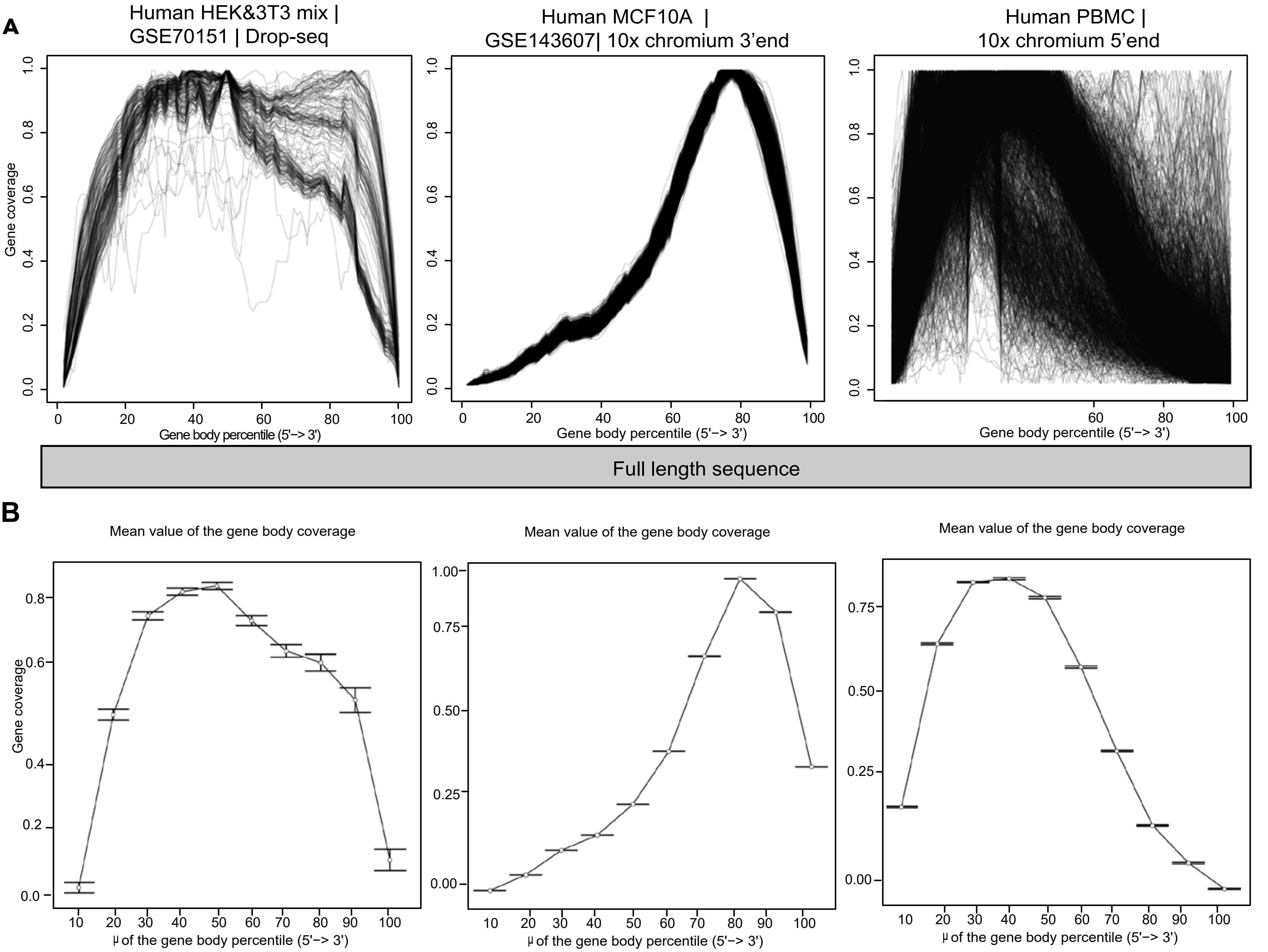

Figure S6: Three human dataset, the GSE70151 from HEK&3T3 cell line generated by Drop-seq, GSE143607 Human MCF10A cell line generated by 10x chromium 3′ end and PBMC generated by 10x chromium 5′ end. (**a**) Distribution of the mapped reads (tags) across the genes. Each panel shows gene body coverage percentile per dataset. The x-axis represents the gene body from 5′ to 3′ end scaled from 0-100, and the y-axis gene coverage (0-1). Each line represents a single cell. (**b**) Mean calculated for bin size = 10.

Figure S7. Gene coverage skewness among single-cell RNA-Seq protocols using different mESCs, related to Figure 1.

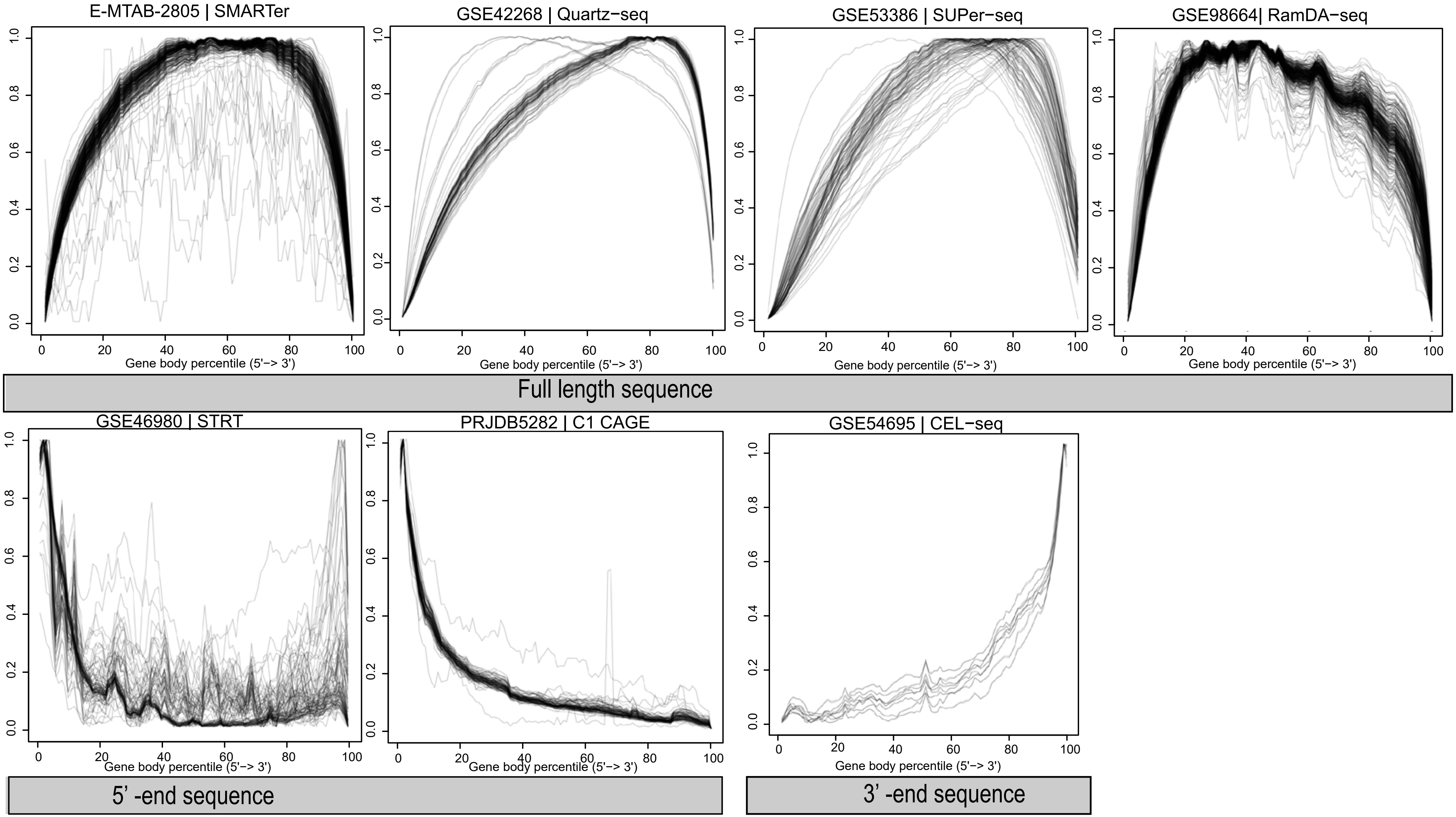

Figure S7: Seven mESCs dataset, top panel dataset generated by fill length sequence protocols (SMARTer, Quartz-seq, SUPer-seq, and RamDA-seq. Bottom panel dataset generated by STRT, C1 CAGE and CEL-seq.

Figure S8. Gene body coverage of typical and skewed cells as obtained by SkewC using Auto Alpha, related to Figure 5.

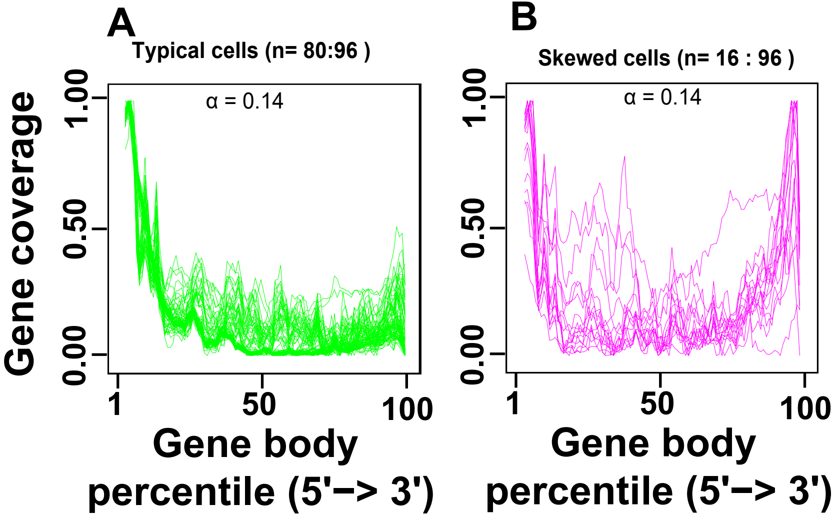

Figure S8: The dataset GSE46980 generated by STRT protocol. Panel (**a**) typical cells and panel (**b**) skewed cells.

Figure S9. Heatmap of the top100 genes between typical and skewed cells, related to Figure 5.

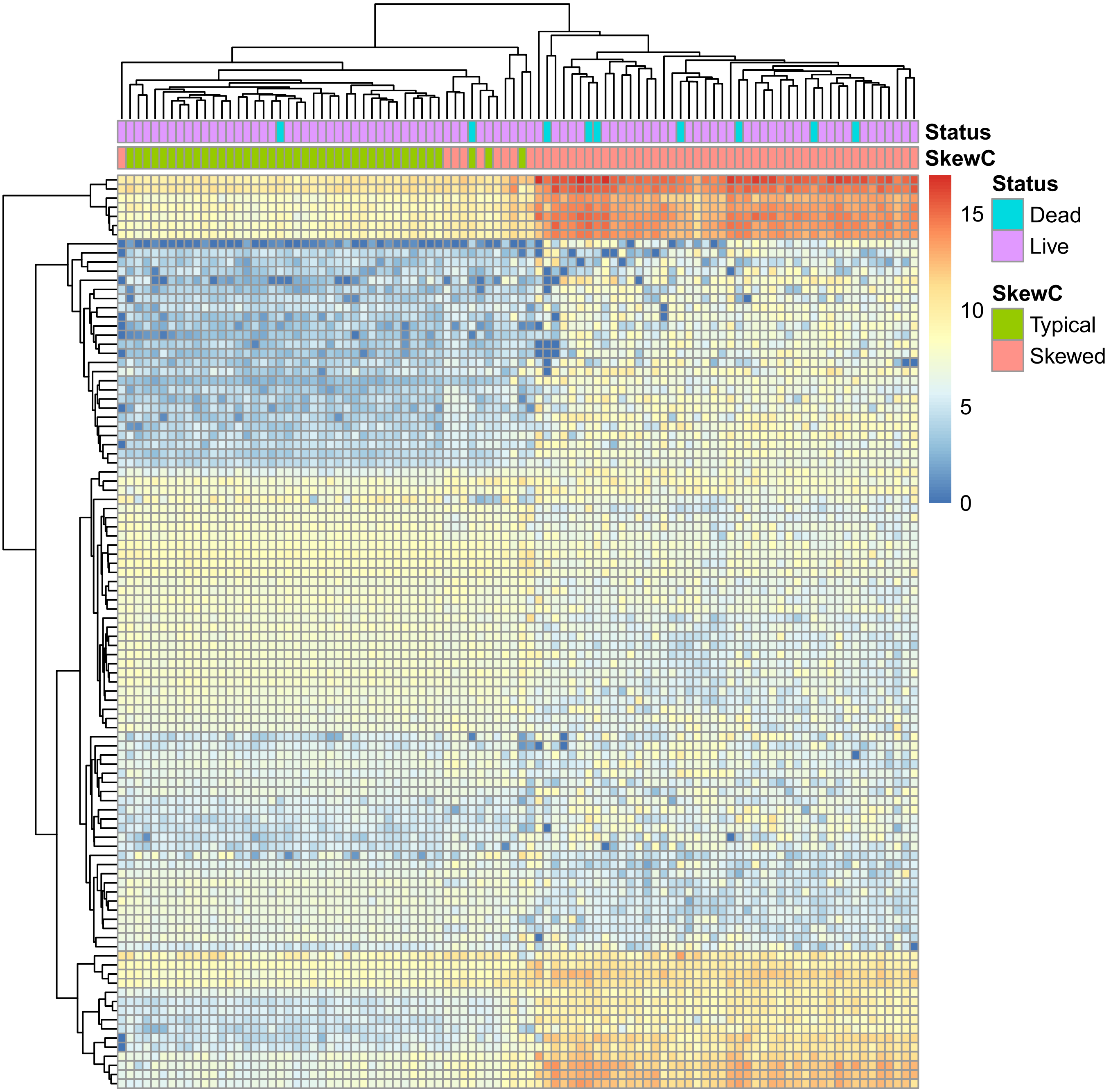

Figure S9: Heatmap of the top100 expressed genes between typical and skewed cells. from GSE46980.
